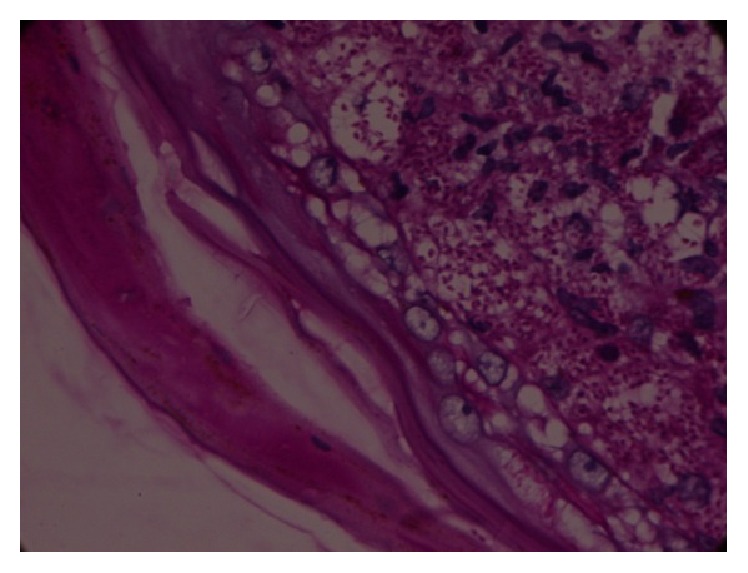# Disseminated Histoplasmosis with Oral Manifestation in an Immunocompetent Patient

**DOI:** 10.1155/2017/1323514

**Published:** 2017-01-31

**Authors:** Debopriya Chatterjee, Aishwarya Chatterjee, Manoj Agarwal, Meetu Mathur, Setu Mathur, R. Mallikarjun, Subrata Banerjee

**Affiliations:** ^1^Department of Periodontics, Government Dental College, Jaipur, India; ^2^SMS Dental Department, SMS Medical College, Jaipur, India; ^3^Department of Endodontics, Government Dental College, Jaipur, India; ^4^Department of Endodontics, Rajasthan Dental College, Jaipur, India; ^5^Department of Prosthodontics, AB Shetty Dental College, Karnataka, India; ^6^Department of Medicine, SMS Medical College, Jaipur, India

## Abstract

A case of disseminated histoplasmosis (DH) in a 60-year-old female patient is reported from Jaipur, Rajasthan, India. The patient presented with multiple papules on the skin surrounding the lips, face, torso, trunk, and back. She also complained of growth in the palate. Histoplasmosis was confirmed by biopsy and histopathology of skin and palatal lesions. This case report highlights the presenting features and occurrence of histoplasmosis in nonendemic region in India.

## 1. Introduction

Histoplasmosis is a dimorphic fungus, which grows in the yeast form in infected tissues. It was first identified by Samuel Darling in 1905, hence known as Darling's disease. Infection is mainly caused by inhalation of droppings from infected birds or bats.* Histoplasma capsulatum* mainly infects the lungs and passes asymptomatically to involve the skin and reticuloendothelial system [1].

Clinically, histoplasmosis has been classified as (i) primary acute pulmonary, (ii) chronic pulmonary, (iii) disseminated form, and (DH) occurring in infants, elderly, or immunocompromised patients [2]. In human immunodeficiency virus (HIV) positive patients, 95% of histoplasmosis appears as disseminated infection. Occurrence of disseminated form of histoplasmosis is very rare in HIV seronegative patients [3].

The manifestations of disseminated form of histoplasmosis are fever, weakness, weight loss, hepatosplenomegaly, and mucocutaneous lesions. The oral lesions may occur in any part of the oral cavity and the lesions vary from nodules to painful shallow or deep ulcers [4]. The incidence of oral manifestation is 25–45% in the disseminated form of the disease [3].

Skin lesions range from papules and plaques with or without crusts, pustules and nodules to mucosal ulcers and erosions, molluscum contagiosum-like lesions, acneiform eruptions, erythematous papules, and keratotic plaques [5].

Although worldwide in distribution, in India, histoplasmosis seems to be prevalent in the Gangetic delta. Panja and Sen reported the first case of disseminated histoplasmosis from Calcutta in 1954 and since then individual cases have been reported from various states, mostly from West Bengal. Among the forms of histoplasmosis reported from India, disseminated histoplasmosis is the rarest [1].

## 2. Case Report

**Figure 1:**
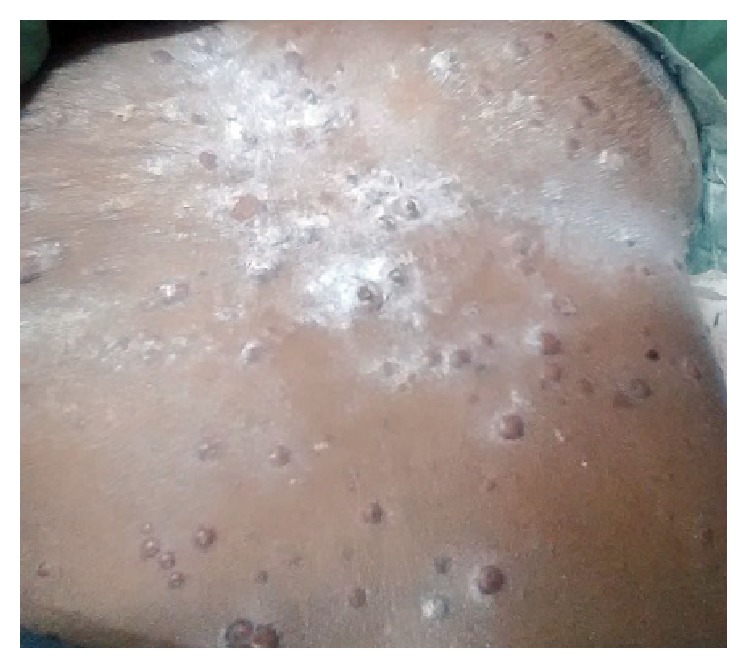


**Figure 2:**
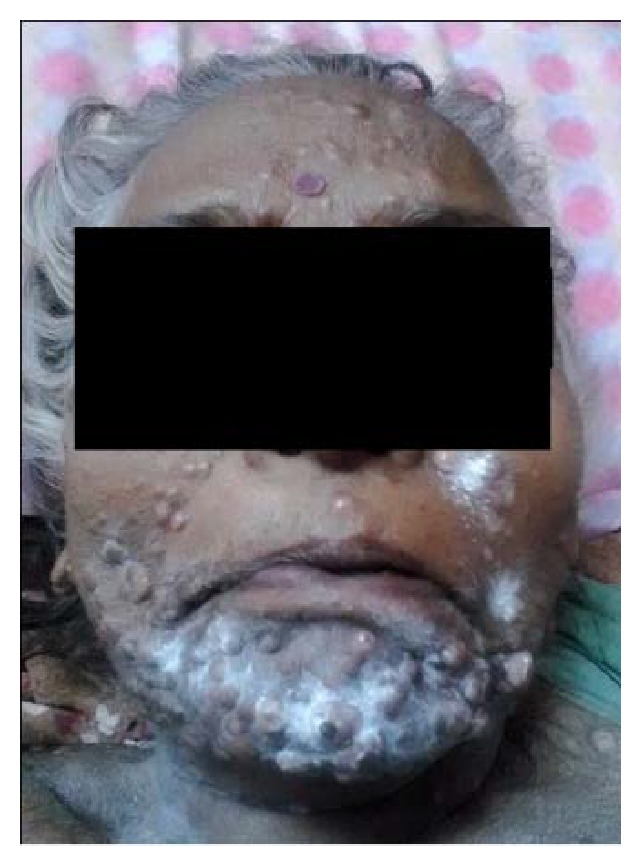


**Figure 3:**
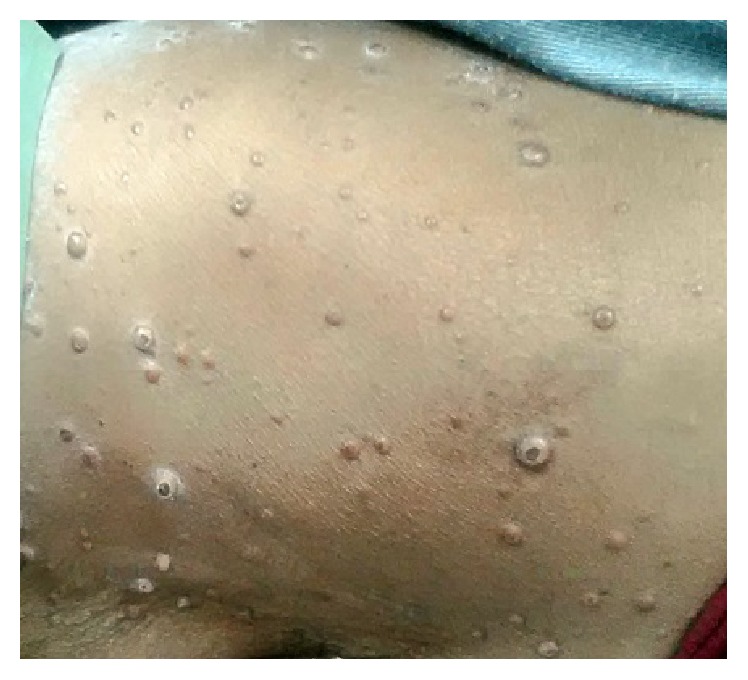


A 60-year-old female was referred from a private practitioner to Dental Department of SMS Medical College Jaipur, with swelling of lower limbs and abdomen for past 3 months. She also complained of difficulty in breathing during exertion. Patient did not have any significant familial history. At presentation, the patient had skin warts which were generalized and tender [Figures 1, 2, and 3]. Clinical examination revealed distended abdomen with splenomegaly and hepatomegaly.

**Figure 4:**
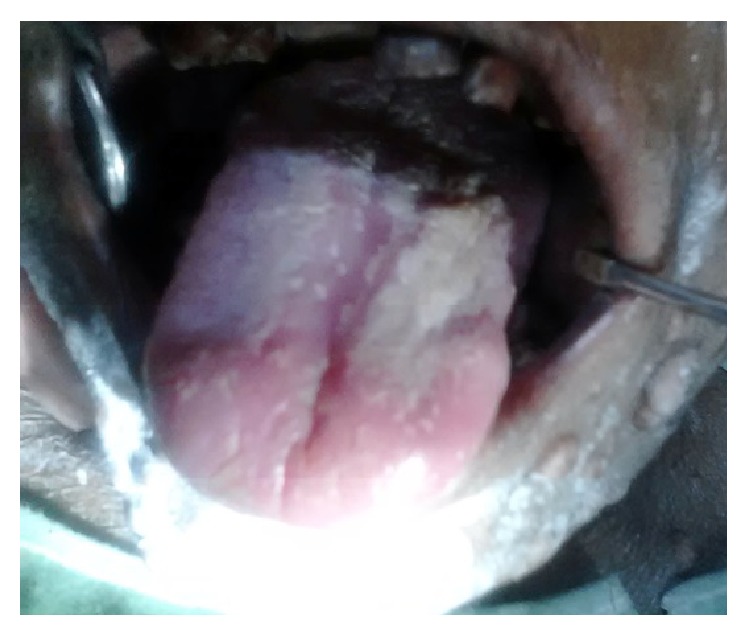


**Figure 5:**
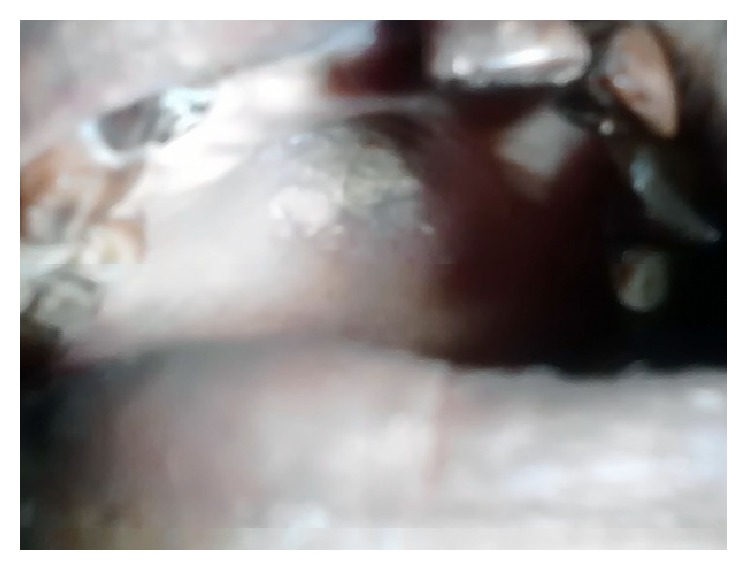


Oral examination revealed ulcerated and necrotic lesions located on the labial mucosa, dorsal surface of tongue, and hard and soft palate [Figures 4 and 5]. The lesions were covered by a pseudomembrane and were painful to palpation. Bilateral submandibular lymphadenopathy was noted. Extraoral examination revealed multiple nodular lesions on the chin, face, and the lips, which were tender to palpation. Multiple nodules were seen on the ventral surface of the forearm and dorsal aspect of thigh. There was a rise in local temperature of the nodules in comparison to the surrounding skin. A firm consistency was felt while palpating these lesions.

Routine investigations revealed random blood sugar was 130 mg/dl (normal range 79–140 mg/dl). Serum creatinine was 1 mg/dl. Serum urea was 30 mg/dl (normal range 15–39 mg/dl). Serum albumin was 3.2 g/dl (normal range 3.5–5.0 g/dl). Serum triglycerides levels were 189 mg/dl (normal range 36–175 mg/dl). Serum VLDL was 38 mg/dl (normal range < 35 mg/dl), haemoglobin was 7.9 gm/dl (normal range 14.0–18.0 gm/dl), and platelet count was 1 lakh/ml (normal range 1.4–4.4 lakh/ml). Levels of urea, creatinine, bilirubins, alkaline phosphatase, and cortisol were normal. Urine examination revealed protein was positive; RBC and pus cell count was 2–4.

The X-ray of the paranasal sinuses, chest, and abdominal ultrasonography did not demonstrate alterations.

Lab investigation revealed no malarial parasites in peripheral blood smear. Febrile agglutination tests for typhoid, brucellosis, and infectious mononucleosis were negative. Antibodies to HIV were negative.

An incisional biopsy of the palatal lesion showed the presence of epithelioid cell granulomas in the connective tissue with numerous histiocytes, many of which formed multinucleated giant cells. The cytoplasm of the histiocytes showed the presence of small round to oval basophilic bodies surrounded by a clear halo, which is the characteristic feature of* Histoplasma capsulatum*.* H. capsulatum*-like yeasts were demonstrated by Periodic Acid Schiff stain. Biopsy of the skin revealed the same histopathologic features [Figure 6].

Antifungal therapy was started with intravenous liposomal amphotericin B at 0.7 mg/kg/day administered for 15 days. Patient's respiratory symptoms showed marked improvement. Oral and cutaneous lesions showed signs of remission. The patient was followed up for 6 months after cessation of therapy, but there was no recurrence. The treatment was tolerated well, with no side effects.

## 3. Discussion

*H. capsulatum* is an intracellular organism. The target organ is reticuloendothelial system and involving the spleen liver, kidney, and CNS.* H. capsulatum* exists as a saprophyte in nature and found in soil, particularly when contaminated with chicken feathers or droppings. By airborne route the spores are infectious to humans [4].

Histoplasmosis is seldom reported from India, due to its varied clinical presentation and lack of awareness amongst dermatologists. Panja and Sen first reported histoplasmosis from India in 1959.* Histoplasma capsulatum* is endemic in certain North Indian states like West Bengal, where a study showed a prevalence of skin positivity of 9.4% to histoplasmin antigen. There are a few sporadic case reports from South India as well [6].

The clinical features simulate other systemic febrile illnesses and most of the times the initial diagnosis is either tuberculosis or malignancy. When it is involving the oral cavity, the most commonly involved sites are tongue, palate, buccal mucosa, gingiva, and pharynx and the differential diagnoses should include squamous cell carcinoma, hematologic malignancy, tuberculosis, other deep fungal infections, oral lesions of Crohn's disease, necrotizing sialometaplasia of the palate, and chronic traumatic ulcers. The palatal ulcers present in our case report resembled squamous cell carcinoma and also the patient had respiratory symptoms similar to tuberculosis [7].

Biopsy of a mucosal or cutaneous lesion might be the most rapid method of arriving at a specific diagnosis of disseminated histoplasmosis [8]. The spores of* H. capsulatum* are visualized in sections stained with hematoxylin and eosin and special stains like Periodic Acid Schiff (PAS).

Antifungal medications are used to treat severe cases of acute histoplasmosis and all cases of chronic and disseminated disease. Amphotericin B is still the drug of choice for disseminated histoplasmosis. For patients who cannot tolerate amphotericin B, itraconazole is an effective and alternative therapy and it may be given as a prophylaxis for patients with advanced HIV infection [9]. Histoplasmosis has been reported in immunocompetent and immunocompromised individuals with the disseminated forms being more common in the latter group [1].

To the best of our knowledge, only one case [10] of disseminated histoplasmosis with oral manifestation has been reported in immunocompetent individual from India.

## 4. Conclusion

Progressive disseminated histoplasmosis is a rare entity among immunocompetent individuals from nonendemic regions. Subjects from endemic regions and around livestock do report occurrences of the disease sporadically. The presenting features of exanthema of the skin and enanthema in the oral cavity are reported for the first time from a nonendemic region in an immunocompetent individual. Histoplasmosis as a differential diagnosis should be kept in mind when diagnosing cases with similar presentations. Early diagnosis and prompt treatment provide alleviation of symptoms and a favourable outcome. However, late presentation at end stages does not respond to medications and may lead to loss of life.

## Figures and Tables

**Figure 6:**